# High cloud coverage over melted areas dominates the impact of clouds on the albedo feedback in the Arctic

**DOI:** 10.1038/s41598-019-44155-w

**Published:** 2019-07-02

**Authors:** Min He, Yongxiang Hu, Nan Chen, Donghai Wang, Jianping Huang, Knut Stamnes

**Affiliations:** 1 School of Atmospheric Sciences, Guangdong Province Key Laboratory for Climate Change and Natural Disaster Studies; Southern Marine Science and Engineering Guangdong Laboratory(Zhuhai), Sun Yat-sen University, Zhuhai, 519028 China; 2 Stevens Institute of Technology, Hoboken, New Jersey 07030 USA; 3 NASA Langley Research Center, MS420, Hampton, Virginia 23681-2199 USA; 4 Lanzhou University, Lanzhou, 730000 China

**Keywords:** Atmospheric dynamics, Cryospheric science

## Abstract

Warming in the Arctic is larger than the global average. A primary reason for this Arctic Amplification is the albedo feedback. The contrasting albedo of sea ice and dark melted surface areas is the key component of albedo feedback. Cloud coverage over the changing surface and the response of the clouds to the changing surface conditions will modify the change in planetary albedo when sea ice melts. Space-based lidar measurements provide a unique opportunity for cloud measurements in the Arctic. The response of clouds to the changing sea ice concentration was directly observed. Based on CALIPSO satellite observations of cloud properties, this study found that cloud coverage in ice-free regions in the Arctic linearly increased with the area of ice-free water during the melt seasons in the past 10 years, while sea ice coverage varies significantly year-to-year. The observations suggest that when sea-ice retreats, cloud fraction of the ice-free region remains fixed at nearly 81%. The high cloud coverage over melted areas significantly reduces the albedo feedback. These results indicate that space-based lidar cloud and surface observations of the Arctic can help constrain and improve climate models.

## Introduction

Given the initial forcing due to the increase in greenhouse gases, through internal feedbacks the climate system adjusts itself to a new equilibrium state. The basic adjustment is higher Surface Air Temperature (SAT). Due to several feedback mechanisms, the SAT increase in the Arctic is 2–4 times larger than the global mean^1–3^. This phenomenon, called the Arctic Amplification, has been confirmed by observations^4^ as well as model simulations^3,5^.

The mechanisms responsible for the Arctic Amplification include: Albedo feedback due to changes in sea ice extent^6^, atmospheric and oceanic heat transports: transport of heat flux that warms the polar area^7^, cloud cover and water vapor that alter the longwave irradiance to the surface^8^, soot on snow, increased black carbon aerosol concentrations^9^. A positive feedback acts to amplify the initial perturbation such as the increasing temperature resulting from extra emission of carbon dioxide, while a negative feedback tends to dampen it. The quantitative contributions of the feedbacks are still being debated. By applying the coupled feedback response analysis method, model assimilation results show that the albedo feedback is the major contributor to the Arctic Amplification^10^, while the cloud feedback is the second largest contributor^5^. However, using the radiative kernel technique, Pithan *et al*.^3^ showed that the dominant contributor is the temperature feedback (low latitude areas emit more longwave radiation to space than the polar areas for the same temperature increase because low latitude areas have a higher basic temperature), and that the albedo feedback is the second largest contributor. Model simulations indicate that the Arctic Amplification may still occur without the albedo feedback^2^.

With the increase in SAT over the last several decades the sea ice extent in the Arctic has retreated significantly^11^. The minimum sea ice extent, in September, has a trend of −11.4% per decade from 1979 to 2007 and the decline is accelerating^12^. The sea ice has been retreating the fastest in the Kara Sea, Barents Sea, and Beaufort Sea regions^13^. As the sea ice melts and ponds form, the albedo decreases greatly. The darker water surface absorbs more solar energy than the sea ice or snow covered surface. The extra absorbed energy will enhance the sea ice melt. This process is well known as the albedo feedback, which is a positive feedback mechanism contributing to the Arctic Amplification. Based on the radiative kernel technique^14^, a quantitative assessment of the albedo feedback’s contribution to the amplification is provided by the model^3^. However, observational assessment of and constraint to the albedo feedback are still lacking.

The climate system continually adjusts itself to the change in sea ice extent. The sea ice layer acts as a thermal and water vapor insulator between the atmosphere and the underlying water. As the ice layer becomes thinner and ponds grow, the exchange of water vapor and heat between the air and ocean increases. On the other hand, the water vapor evaporation will increase as the SAT increases if we assume fixed relative humidity over the open water. Reduction in sea ice will lead to increased heat and moisture fluxes from the ocean surface^15^. A loss in sea ice extent of 0.1 Mkm^2^ is estimated to result in a 10–20% increase in moisture content in the Arctic^16^. Arctic regions with more sea ice are found to have a smaller cloud fraction and a smaller cloud liquid water content^17^. On average, clouds cool the Arctic from February to November through the shortwave forcing and warm the Arctic all year around through the longwave radiative forcing^18^. Detailed radiative effects in polar areas are complex and may vary from negative to positive due to the high frequency of temperature inversions. The response of clouds to sea ice retreat may have significant impacts on the radiative energy balance in polar regions. Because of the comparable albedo of sea ice and cloud, a change in cloud cover in response to an increase in open water during the melt period may significantly affect the radiative energy balance by adjusting the albedo feedback process.

Given an initial perturbation (*δT*_*s*_) to the climate system, the system tends to adjust itself through a variety of feedback processes. For example, the net TOA flux ($\bar{R}$) is determined by the water vapor abundance, temperature, cloud properties, surface albedo and so on for a given place and time. The initial perturbation may induce changes in all of the feedback parameters. The total perturbation in the TOA flux can be expressed as a summation of partial radiative perturbations from each feedback parameter. A feedback parameter for each variable *X* can be expressed as the radiative kernel and climate response pattern^14^. The radiative kernel is the ratio of TOA flux perturbation and the change in the feedback parameter such as the anomaly of sea ice concentration (SIC for short). The radiative kernel can be used to assess climate feedbacks consistently across models. For example, the contributions from a variety of feedbacks to the Arctic Amplification were examined by comparing radiative kernels consistent results produced by climate models^3^. However, radiative kernels are generally obtained from model simulations. The anomalies of SIC and radiative forcing at TOA provide us with an opportunity to calculate the radiative kernel from observations obtained by space-based sensors.

Due to sea ice (with partial snow cover), the Arctic Ocean has an albedo of about 0.65^19^. In stark contrast, a liquid water surface is quite dark with an albedo of about 0.1^19^. Hence, during the period of sea ice melt, the albedo changes from 0.65 to a value approaching 0.1 implying that the surface will absorb more solar energy. The extra energy further enhances the SAT and accelerates the sea ice melt. However, the albedo of clouds is slightly smaller than but comparable to the albedo of sea ice. The planetary (TOA) albedo is due to reflection from both the surface and the atmosphere. On a global scale, the atmospheric reflection contributes about 88% of the planetary albedo^20^. In the Arctic, the response of clouds to the change in sea ice extent, snow cover, and melt pond fraction will impact the TOA albedo, and hence the final contribution of the albedo feedback to the Arctic Amplification.

Both sea ice and clouds play essential roles in the climate system, and their longterm variations are the primary indicators of climate change^21^. The climate effects of sea ice and clouds have been intensively studied for several decades. Recent studies confirmed that the sea ice retreat has had a significant impact on the Arctic cloud cover. Arctic clouds can be influenced by the large scale atmospheric circulation, near surface stability and surface conditions^22^. The cloud properties differ significantly between meteorological regimes^23^. Synoptic regimes, such as the warm advection, cold advection, can impact clouds at different heights and influence the date of sea ice melt onset^24^.

Clouds and sea ice have a significant covariance under stable conditions^17^. Liquid clouds respond to the sea ice variability except during summer^25^. Results obtained from passive sensors show that low clouds can form over newly formed open water during early fall^22^. Based on simulations, Abe *et al*.^26^ found that the cloud fraction increased with the retreat of sea ice and that the increase in cloud cover would enhance the longwave warming. The minimum sea ice extent in 2012 was followed by a decrease in cloud cover in winter^27^, which may have led to a cooling of the surface and an increase in sea ice extent the following year. Hence, changes in cloudiness can potentially influence the sea ice extent^28^.

In this paper, we investigate the response of summer and early fall cloud cover to changes in sea ice extent and its impact on the shortwave and longwave radiation budget. Due to frequent temperature inversions and small temperature contrast between the surface and cloud in the Arctic, passive sensors have limited ability to detect clouds in the polar regions^29^. By applying active sensors, combined CALIPSO and CloudSat observations can overcome the shortcomings of cloud products provided by passive observations in the polar regions. To this end CALIPSO and CloudSat measurements are used to study the cloud-sea ice-climate feedback: the feedback due to sea ice retreat and the response of clouds to the sea ice retreat.

## Results

### The cloud fraction and SIC anomaly

In the context of global warming and significant sea ice retreat, both sea ice thickness and concentration show a regionally dependent variability^30^. Several factors contribute to the variation in SIC and their impact depends on location. Focusing on the sea ice coverage, the Arctic can be divided into 3 different sections: the open water section, the permanent ice covered section, and the transitional section. Due to oceanic heat transport in the Atlantic section of the Arctic, the Greenland Sea, the Fram Strait, the Barents Sea, and the Kara Sea are open most of the year. The open water section is defined by the area with average SIC less than 20%. Surrounded by the continents and islands, the relatively closed portion of the Arctic Ocean (which includes the Lincoln Sea, the outer part of the Canadian Archipelago, and part of the Beaufort Sea) has average SIC greater than 90%. The section with average SIC greater than 90% is called “permanent ice covered” section. The transitional section of the Arctic Ocean has SIC varying from 20% to 90% with distinct seasonal variation, and it also exhibits significant inter-annual variations. These SIC variations have a considerable impact on the cloud coverage.

**Figure 1:**
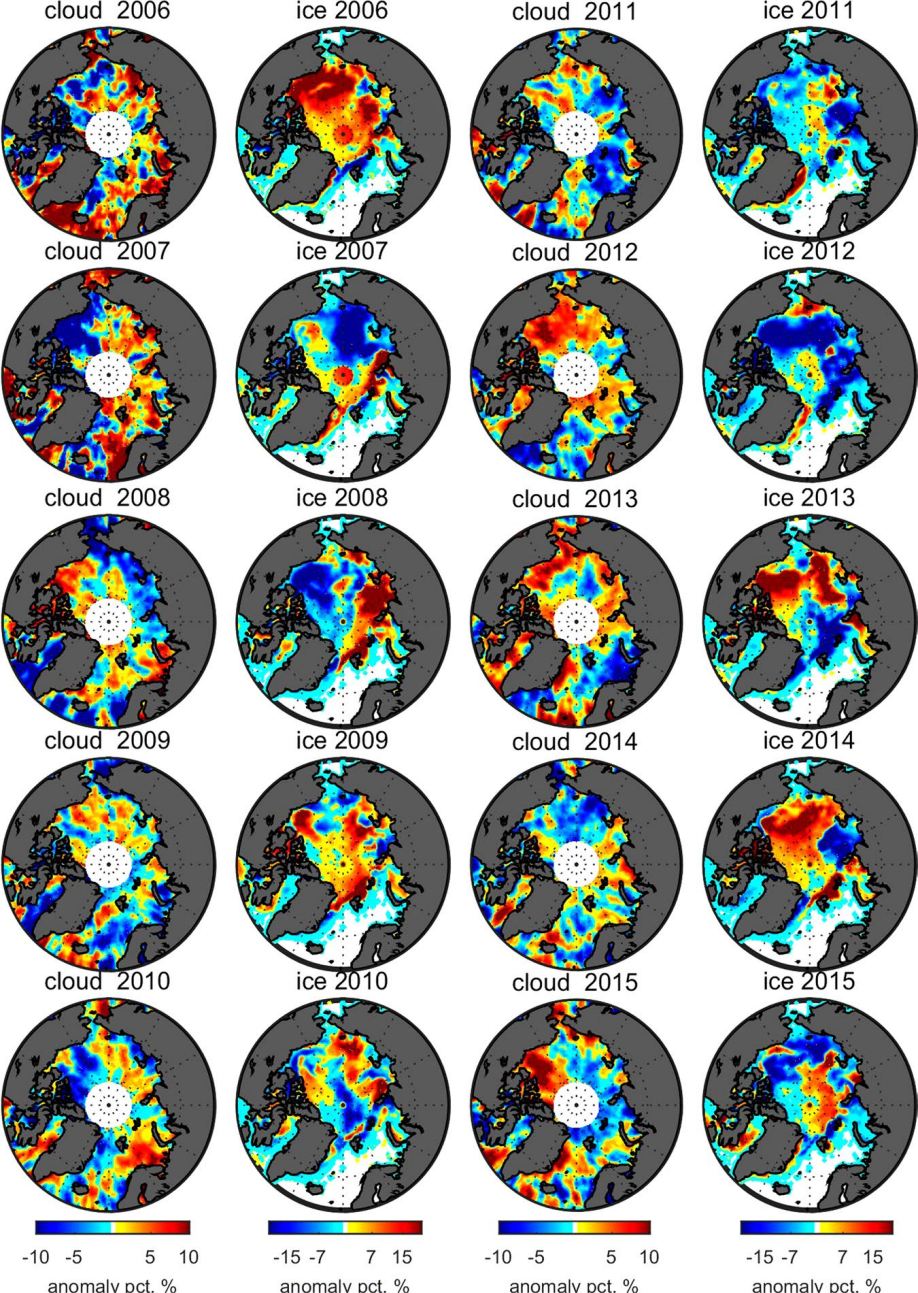
Anomalies (in absolute units) of cloud fraction obtained from CALIPSO observations during the summer and fall seasons (July to October) and sea ice concentration. The anomalies are calculated by subtraction of the 10-year (2006–2015) average. The cloud fraction is the average between August 1 and October 15. Sea ice concentrations are based on the average of the period from July 1 to September 15, which covers melting season.

The mean SIC in the melt season, taken to be from July 1 to September 15 in this paper, is used to represent the sea ice coverage for a given year. Focusing on the anomaly of the mean SIC, calculated by subtracting the areal average from 2006 to 2015, we find that the mean SIC departs from the average, and that the anomalies show regional and inter-annual variations. The SIC anomalies of 2006 and 20015 are shown in Fig. 1 (second and fourth columns). The corresponding anomalies of the cloud fraction are shown in the first and third columns in Fig. 1. The regional distributions of the cloud anomaly are closely related to those of the sea ice anomaly. The reduced sea ice coverage may enhance the evaporation implying that an increase in cloud coverage is expected to be observed^25^. The covariance of the sea ice anomaly and the cloud anomaly confirms the response of cloud cover to the change in sea ice. Based on 10-year observations, the covariance has different patterns. The directions of the cloud anomaly are mostly opposite to those of the sea ice anomalies for most years and regions. For example: the Beaufort Sea area, which has a strong positive sea ice anomaly, shows a negative cloud anomaly in 2006. The contrasting cloud and ice anomalies are distinctly regionally dependent in 2007, 2008, 2012, and 2014. Only part of the transitional section shows opposite cloud and sea ice anomalies in 2006, 2009, 2010, 2011, 2013, and 2015. Areas that have negative sea ice anomalies do not always have positive cloud anomalies and vice versa. For example, both the cloud and sea ice have positive anomalies in the East Siberian Sea area in 2009 and 2010, where the cloud and sea ice anomalies are negative in the Laptev Sea area in 2011 and around the Sevemaya Zemlya area in 2012.

On average, the uncertainty of the cloud fraction is 5% at the 95% confidence level in absolute units. In seven of ten years, the maxima of the cloud anomaly can exceed the uncertainty. However, the cloud anomalies in 2009, 2011, and 2014 are not significant as their values are smaller or comparable to the uncertainty. The cloud anomalies are heterogeneous even over a large portion of the ice anomaly area. In 2012, the ice anomaly is negative from the Beaufort Sea to the Laptev Sea area where the cloud anomaly is positive but with some negative spots.

**Figure 2:**
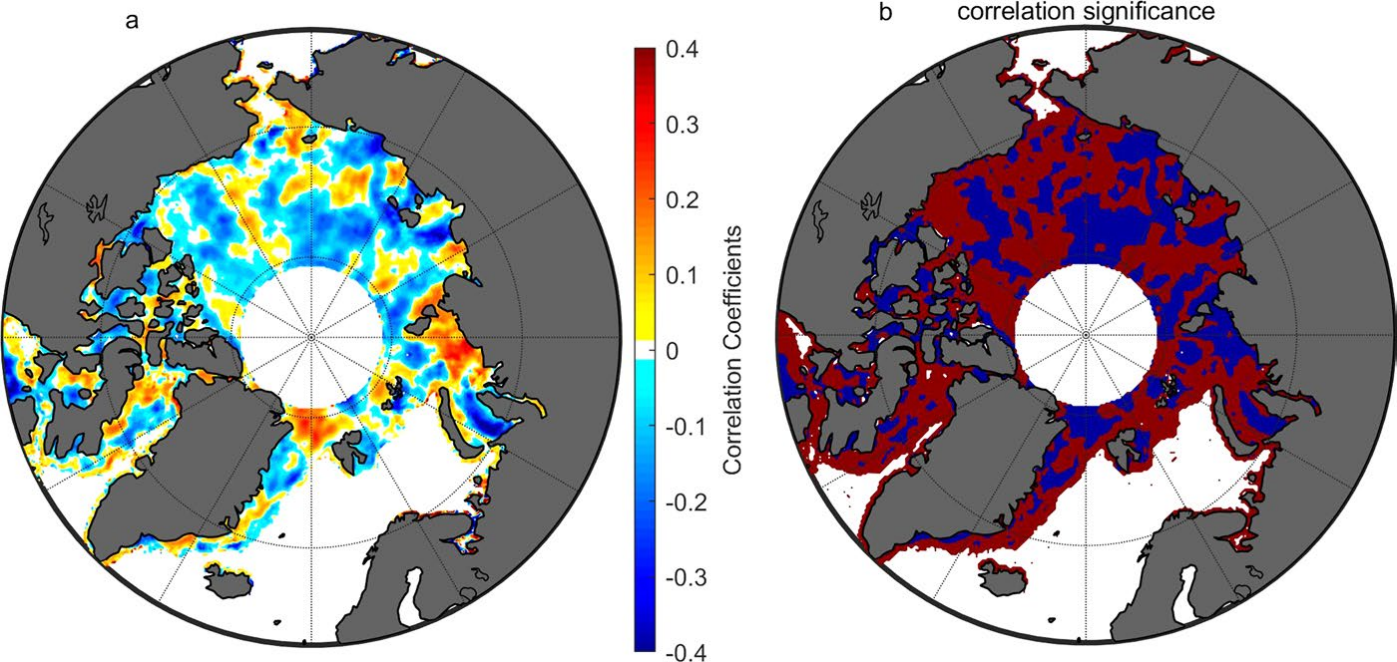
(**a**) Map of Pearson correlation coefficient between the cloud and SIC anomalies. The white areas have insufficient samples to allow calculation. (**b**) shows the significance of the correlation relation. The blue color means the relation is true at the 95% confidence level. The red color means the probability is less than 95% for the correlation to be true. Both (**a**,**b**) are based on the sampling elements of the average of cloud anomaly and SIC anomaly for a 9-day period and in the 3° (latitude) by 8° (longitude) box.

Based on the ten years of observations, the Pearson correlation coefficient and the correlation significance are shown in Fig. 2 (subfigure **a** for the correlation and **b** for the significance). About 40% of the area does not show significance in the correlation. The correlation coefficients are within the range between −0.4 and 0.4. Most of the area shows negative correlation, which means that cloud cover increases as the sea ice retreats. A part of the Arctic Ocean shows a positive correlation. The significant negative area includes the East Siberian Sea and the Beaufort Sea. The most significant positive area is the Kara Sea.

Overall, the geographic distribution of the covariance between clouds and sea ice confirms the response of clouds to the sea ice loss^22,25^ once again. The correlation coefficients between the sea ice anomaly and cloud anomaly are negative in most areas but positive in some areas. The region of negative correlation is more significant than the positive area. The nonuniform regional covariance reveals that the response maybe influenced by other factors such as atmospheric circulation. The significant but nonuniform response of clouds to the sea ice retreat may influence the albedo feedback. The time lag between cloud formation and the enhancement of evaporation is considered in the investigated periods of sea ice and cloud correlation. The sea ice anomaly is based on the average over the melt period from July 1 to September 15. The cloud anomaly is based on the average over the period from August 15 to October 15 which is later than that of the sea ice.

### Effects on the TOA albedo

**Figure 3:**
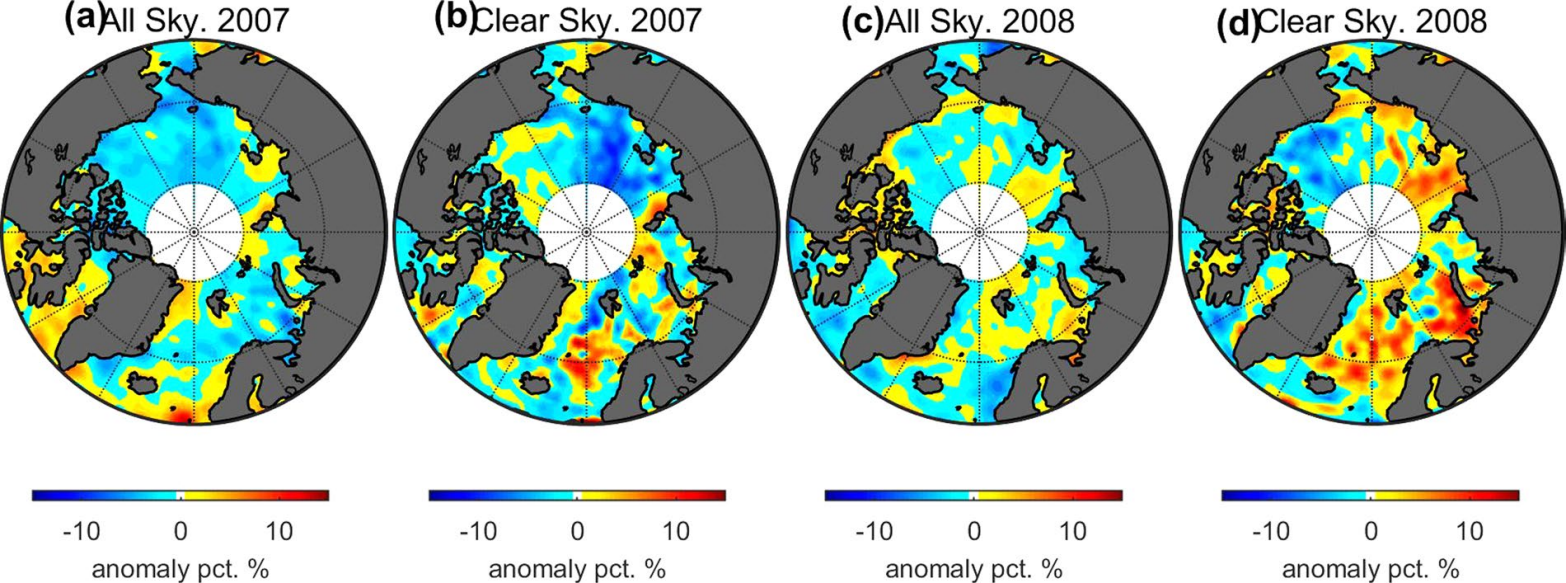
Albedo anomalies (absolute values) of all sky (left column) and clear sky (right column) conditions during the investigated period. All sky includes both clear and overcast sky condition. The anomalies were calculated by subtraction of the 4-year average. (**a**) The albedo anomaly of all sky for 2007, (**b**) the albedo anomaly of clear sky for 2007. (**c**) Same as (**a**) but for 2008, (**d**) same as (**b**) but for 2008.

As the surface changes during the melt period from snow-covered sea ice to partially snow- melt pond-covered sea ice to totally open water, the albedo of this heterogenous surface will decrease. Because the cloud albedo lies between the albedo of snow and the albedo of dark open water, an increase in cloud cover due to loss of sea ice coverage is expected to partly compensate for the associated albedo decrease and tend to restore the TOA albedo during the sea ice melt period to the pre-melt value. The planetary albedo is determined by reflection from both the surface and the atmosphere. Under clear sky conditions, the TOA albedo is primarily determined by the surface condition. The albedo anomalies, calculated by subtracting the areal average from 2007 to 2010, under clear sky conditions, shown in Fig. 3b: 2007 and Fig. 3d: 2008, have the same direction as that of the SIC anomalies (Fig. 1a,c). For 2007, the East Siberian Sea and Kara Sea areas have a negative albedo anomaly (−10%), while the Beaufort Sea area has a slightly positive anomaly (+2%). For 2008, the TOA albedo has a positive anomaly (+10%) in the Kara Sea area and a negative anomaly (−8%) in the Beaufort Sea area. The distribution of the anomaly directions is consistent with the distribution of the SIC anomaly shown in Fig. 1.

However, the TOA albedo anomaly for all sky conditions, shown in (Fig. 3a: 2007 and Fig. 3c: 2008), is much smoother than that of the clear sky. The TOA albedo anomaly is reduced to about ±4% for both 2007 and 2008. For 2007, the Laptev Sea and East Siberian Sea have a smaller SIC and also exhibit a negative anomaly of about −10% in the clear sky TOA albedo. When the effects of clouds are taken into account, the TOA albedo anomaly of all sky conditions in this area is reduced to −4%. The increase in cloud amount due to the sea ice retreat can recover the TOA albedo by about 6%. The same area (Laptev Sea) but for 2008, has a positive SIC anomaly and shows a TOA albedo anomaly of 12% under clear sky conditions. The presence of clouds reduce the TOA albedo anomaly to about 3%. Fewer clouds can restore the TOA albedo by about 9%.

Under clear sky conditions, the TOA albedo is determined by the surface and the albedo anomalies are coincident with the anomaly of SIC. Under all sky conditions, the TOA albedo is determined by clouds and the surface. The modification of cloud cover in response to sea ice retreat will affect the TOA albedo anomaly.

### Radiative effects at TOA

Clouds primarily scatter (and reflect) shortwave radiation and primarily absorb longwave (terrestrial) radiation. Clouds re-emit the absorbed radiation at the effective cloud temperature, which (except for temperature inversions) is usually lower than the surface temperature. The cloud is negatively related to the SIC at part of the Arctic area. When sea ice retreats, more clouds are expected to form and have radiative effects: (i) increase the solar energy reflected to space and thereby cool the atmosphere (surface), and (ii) reduce the outgoing longwave radiation which warms the surface. The net radiative effect of clouds may change from warming to cooling due to frequent temperature inversions and low solar elevations in the polar regions.

**Figure 4:**
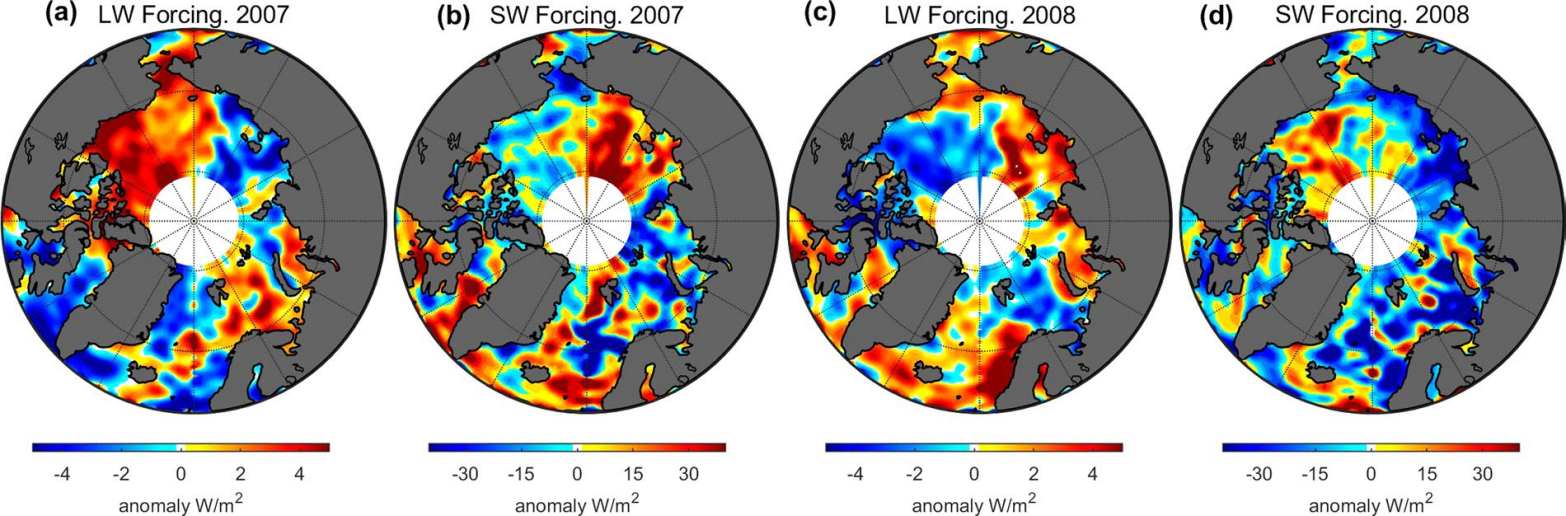
Cloud forcing anomaly of shortwave and longwave upward irradiance at the TOA during the investigated period. (**a**) The anomaly of longwave radiative forcing for 2007; (**b**) the anomaly of shortwave radiative forcing for 2007; (**c**) same as (**a**) but for 2008; (**d**) same as (**b**) but for 2008.

The cloud forcing anomalies of the TOA reflected shortwave irradiance are shown in Fig. 4b: 2007 and Fig. 4d: 2008. For 2007, the cloud fraction has a positive anomaly of 6–8% (in absolute unit) in the Kara Sea and Laptev Sea areas. This 6–8% cloud fraction anomaly results in up to a 40 W/m^2^ anomaly in shortwave TOA irradiance in the Kara Sea and Laptev Sea areas. The Beaufort Sea area, which has a 8–10% smaller cloud fraction than the longterm average, shows a negative anomaly of 4–10 W/m^2^ in shortwave TOA irradiance.

For 2008, based on the observed cloud fraction and radiative flux, the cloud fraction shows a positive anomaly of 2–6% in the Beaufort Sea area, which implies an extra short wave radiative energy of 20–40 W/m^2^ reflected to space compared to the longterm average (see Fig. 1). A negative cloud fraction is found in the Laptev Sea and East Siberian Sea (about – 4–6% in absolute units), where the cloud forcing anomaly of the TOA shortwave irradiance exceeds 30 W/m^2^.

In addition to the reflection of sunlight, clouds also act as effective longwave absorbers/emitters that redistribute the longwave radiation. More cloud cover means less longwave radiation emitted to space and vice versa. Figure 4 shows the cloud forcing anomaly of the TOA longwave radiation. For 2007, the cloud fraction has a strong negative anomaly (over 10%) in the Beaufort Sea area and a moderately negative anomaly (4–6%) in the Chukchi Sea. Corresponding to these reductions in cloud amounts, these areas have strong/moderate (1–5 W/m^2^) positive anomalies of the longwave radiative forcing. The cloud forcing anomalies of the TOA longwave radiation are negative (over −5%) in the Laptev Sea area which has a positive cloud fraction anomaly (2–5%).

For 2008, the cloud fraction has a negative anomaly (−4–6%) in the Laptev Sea which shows a strong positive cloud forcing anomaly (2–4%) of the TOA longwave irradiance. A positive cloud fraction anomaly (2–5%) occurs in the Beaufort Sea which has up to −5 W/m^2^ negative cloud forcing anomaly of TOA longwave radiation. A positive cloud fraction anomaly occurs in the Laptev Sea and East Siberian Sea in 2007 and in the Beaufort Sea in 2008 where a total (shortwave and longwave) extra irradiance of up to 35 W/m^2^ escapes to space. A negative cloud fraction anomaly occurs in the Beaufort Sea in 2007 and in the Laptev Sea in 2008 where a total irradiance of 10–25 W/m^2^ escapes to space. The strength of ice albedo feedback in the climate system can be represented by a feedback parameter defined as^14^:

1 $$\lambda =\frac{\delta R}{\delta \sigma }\frac{\delta \sigma }{\delta {T}_{s}}$$

where *δR* is the change of TOA cloud radiative forcing, *δσ* is the change of the sea ice loss given a perturbation in the surface temperature *δT*_*s*_. The first part of the right hand side in Eq. (1) is defined as the radiative kernel. We use the anomaly of sea ice concentration to represent the change of surface. Thus the radiative kernel of ice albedo feedback is estimated through the observation with average of −0.46 ± 0.90 Wm^−2^ percent^−1^ for the shortwave radiation and +0.14 ± 0.087 Wm^−2^ percent^−1^ for the longwave radiation. These results mean that a one percent decline in SIC will result in an additional shortwave forcing of −0.46 Wm^−2^ and longwave forcing of +0.14 Wm^−2^ due to changes in cloud cover.

The neutral cases which have anomalies less than 8% are discarded to avoid the small number problem which means divided by a small number to produce unstable cases. The cases in the distribution range greater than 3 times of deviation are also discarded. These values represent the situation that sea ice anomaly not exceed 20%. Limited by the length of the CERES, CALIPSO, CloudSat, and MODIS (CCCM) merged dataset used in this study (see the “Method” section of details), the radiative kernels are based on observations from 2007 to 2010.

### The cloud fraction over the melted portion of Arctic Ocean

The albedo is determined by the surface type, the clouds above the surface and the response of clouds to the surface change. The response of clouds to the surface change shows regional variability as shown in Fig. 1. The response of clouds to the surface change is expected to influence the albedo feedback. However, the key to the albedo feedback is the change of surface albedo in the process of sea ice/snow melt. Thus clouds over the melted area can impact the albedo feedback directly. In this section, we investigate the cloud coverage over the melted portion of the Arctic and the relation between the average cloud fraction and the sea ice extent.

**Figure 5:**
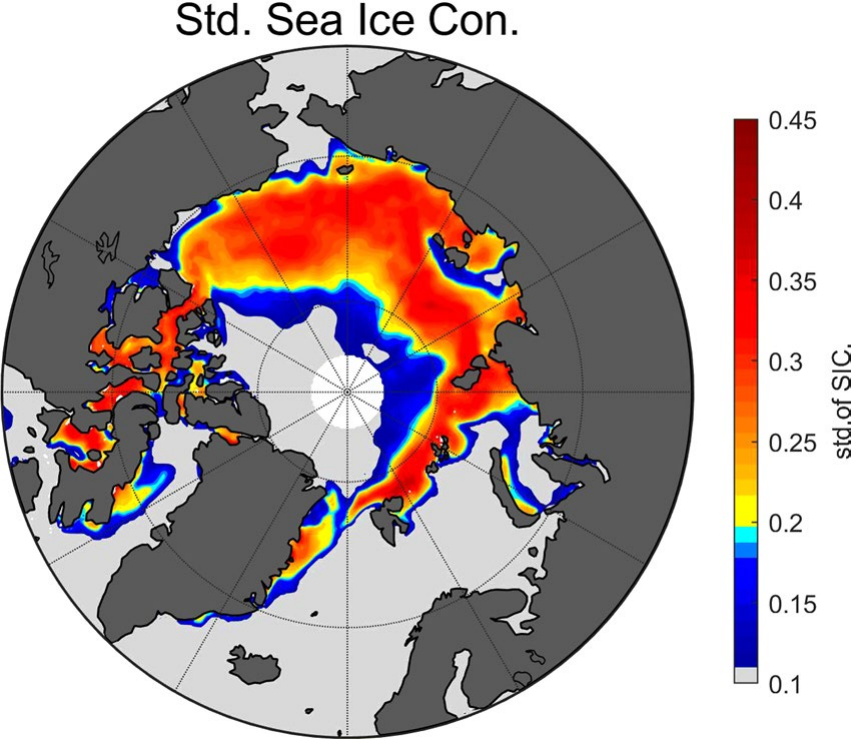
The sensitive zone of sea ice during summer. The colorbar indicates the standard deviation of the average sea ice concentration during the investigated period from 2005 to 2015. The light gray color area has standard deviation of SIC smaller than 0.1. The small variation implies that the topographic type of ocean surface maintains during the investigated period. The outer part of the area that has std of SIC smaller than 0.1 is always open water while the inner part is always covered by sea ice. The area located between 90° East and 120° West, having std values greater than 0.2, is defined as the sensitive zone.

We define a sensitive zone as one having a significant variation in SIC during the time period analyzed: July through October. The closed section of the Arctic, which is surrounded by the continents of Asia and North America and the islands of Greenland, Svalbard, and Franz Josef’s Land, is different from the Atlantic section of the Arctic. The ocean currents have a huge impact on the Atlantic section of the Arctic Ocean which consist of open water most of the time. However, the closed section of the Arctic experiences significant variation in sea ice extent. Figure 5 shows the area of the sensitive zone which has a very large variation in SIC over the investigated time. This sensitive zone is defined as the area located between 90° East and 120° West and with standard deviation (std) of SIC larger than 0.2. The surface in this selected sensitive zone is likely to experience an annual melt- freeze up- cycle during the period of investigation (2006–2015).

A frequently used indicator of sea ice coverage is the minimum sea ice extent which occurs in September. Considering the effect of time evolution of melt and freeze up, we use the average sea ice extent from August 15 to October 15 to examine its relation to cloud cover. On the other hand, to take into account the lag between cloud formation and enhanced evaporation after sea ice melt, the average cloud fraction during the period from August 1st to October 30th was used in this part of study.

**Figure 6:**
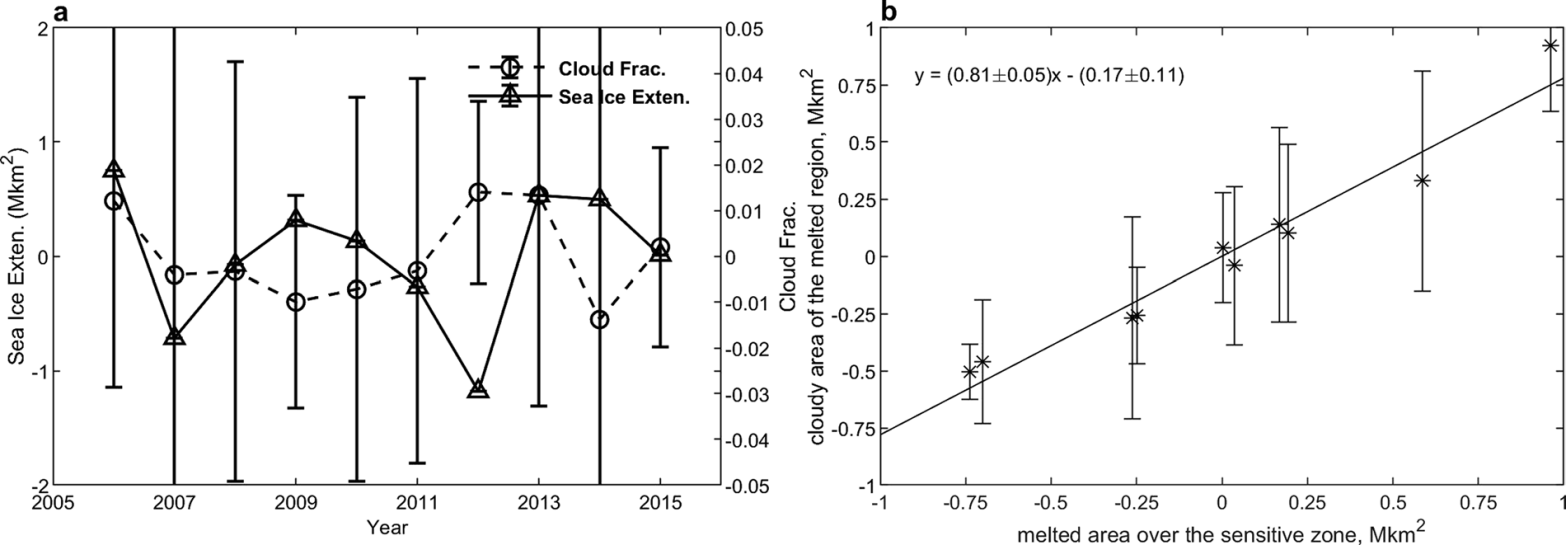
(**a**) Time series of the cloud fraction (triangles ∆) and sea ice extent (circles ○). Both cloud fraction and sea ice extent have been subjected to a linear detrending process. The cloud fraction is calculated from the sensitive zone over the period from August 1 to October 15. The sea ice extent is the north hemispheric average between the August 15 and September 15 when the minimum sea ice extents are included. (**b**) The relation between the cloudy area of the melted Arctic Ocean and the melted area at the sensitive zone. *x*-axis: the melted area in the sensitive zone in Mkm^2^. *y*-axis: the surface area of the melted region covered by clouds. The std of the melted area that covered by cloudy has an average of 0.32 Mkn^2^. The uncertainties of the regression coefficients satisfy the significance of 95%. Linear de-trending has also been applied to the areas. Detrending is used to remove the linear fitting from the average cloud fractions or areas.

The time series of the average cloud fraction (after detrending) and the average sea ice extent (after detrending) during the summer and autumn from 2006 to 2015 are shown in part (**a**) of Fig. 6. We note that the cloud fraction increases as the sea ice extent shrinks, and vice versa. As the sea ice extent increasing, the evaporation and cloud amount reduce. The sea ice anomaly may change from positive to negative in the sensitive zone and so as the cloud anomaly. The fluctuation of the average cloud fraction is 3% in absolute value which is smaller than the uncertainty (5% absolute value). The fluctuation of the sea ice extension is 1.5 Mkm^2^ in absolute value and 21% compared to the minimum sea ice extent in relative value. The correlation coefficients between clouds and SIC change from positive to negative Fig. 2. The response of clouds to the sea ice is only locally significant but not on the average over the sensitive zone. Based on the observations from 2006 to 2015, no significant relation appears between the average cloud fraction and the sea ice extent.

Since the average cloud fraction only show weak relation to the change of sea ice extent, it is expected that a high percentage of melted areas, where pixels have SIC values smaller than 15%, to be covered by clouds. The statistical relation of the melted area and the melted area that is covered by clouds is shown in the right panel (**b**) of the Fig. 6. About (81 ± 5)% of the melted area in the sensitive zone is covered by clouds. The essential of the albedo feedback is the wane of albedo when the surface type change from sea ice to the darker water. From the CALIPSO observation, about 81% of the melted area is covered by clouds. The uncertainty of the melted area is 0.32 Mkm^2^ which equals to the uncertainty of 15% in the cloud fraction. The high coverage over the melted dark water can compensate about 81% of the albedo-feedback in the Arctic Ocean area.

In the process of sea ice melt, the clouds fraction maintains a constant value of 81% over the melted ocean. This phenomenon may indicate that high cloud coverage over the melted ocean dominates the impact of cloud cover on the albedo feedback. The increase in the cloud fraction in the entire sensitive zone is not significant as a function of the sea ice extent. The response to sea ice is only a second order contribution to moderate the change in albedo due to sea ice melt.

## Conclusion and Discussion

Previous studies already show that a warmer Arctic is cloudier and that the corresponding cloud forcing changes from warming the surface to cooling the surface in different seasons^31^. The interactions between the clouds and the surface are complex and play an important role in the Arctic energy balance. This study presents the local character of the damping effect by the clouds in the Arctic climate system. The albedo feedback is one of the most pronounced positive feedback in the polar regions. Clouds can damp this positive feedback: cloud formation can partly compensate for the change in albedo due to the melting of sea ice. The damping mechanism works in two ways. The first one is the high coverage over the melted area. The second is cloud formation as the enhanced evaporation after sea ice melt. Several positive feedbacks such as albedo feedback, temperature feedback, water vapour feedback and so on work in parallel to amplify the SAT increase in the Arctic. The rise in SAT will enhance sea ice melt and reduce the surface albedo. However, the clouds can partly remedy the reduction in albedo which is due to those positive feedbacks.

Based on the lidar observation in Fig. 1, the cloud anomalies reveal a regional response to the SIC anomalies. The cloud anomaly and SIC anomaly show a significant negative correlation in the Beaufort Sea and the East Siberian Sea. The correlation between the cloud anomaly and the SIC anomaly is non-uniformly distributed across the Arctic Ocean with a variation from positive to negative. The covariance of cloud and sea ice is locally significant but with nonuniform distribution. The two anomalies are not necessarily in the same location. For example, the SIC has a negative anomaly at a portion of the West Siberian Sea, where the cloud fraction also shows a negative anomaly for the year 2007. For 2007, the Beaufort Sea area has a slight increase in sea ice extent, but extremely low cloud fraction. The final TOA albedo anomaly is slightly negative.

The observed radiative forcing and SIC anomalies can be used to quantify the radiative kernel. These kernels represent the mean situation for SIC anomalies in range of 0.08 to 0.20. The radiative kernel represents a quantitative assessment of the feedback. A positive longwave radiative kernel means that sea ice loss will reduce upward longwave radiation through extra cloud formation stimulated by the sea ice loss. A positive longwave radiative kernel directly confirms the response of clouds to sea ice retreat.

The cloud fraction shows a likely response to the variation in sea ice extent, but with outliers on a decadal time scale. The fluctuation of the average cloud fraction is within the uncertainty of the cloud fraction^29^ observed by the CALIPSO lidar. The sensitive zone experiences a loop of melt and freeze-up. The sea ice extent has significant change during the period from August to October. The average cloud fraction of the sensitive zone is negatively related to the sea ice extent. This result indicates that the melting sea ice can result in an increase in the cloud amount. The fluctuation in SIC during the decade from 2006 to 2015 is 21% in the sensitive zone over the period from August to October. Because of the opposite anomaly directions at different geographic zones, the average cloud fractions of the sensitive zone only have fluctuation of about 2% but with huge uncertainty. The melted area has a constant cloud coverage of (81 ± 5)%. The albedo feedback is typically explained as the tremendous change in albedo occurring when the surface changes from snow or sea ice to open water. However, the dark melted (open water) areas are frequently covered by clouds. A cloud fraction of 81% over the melted areas can reduce the sea-ice/snow albedo feedback. We can conclude that: the high cloud coverage over the melted water dominates the albedo change in the albedo feedback process. On average, the extra cloud cover stimulated by sea ice loss is of second-order contribution.

Previous studies were aimed at revealing the interactions of cloud cover, sea ice concentration, radiative heating and weather conditions. Weak but significant covariance between cloud cover and sea ice exists in different atmospheric regimes^17,22,25^.

The new insights provided in this paper include the response of clouds to the melting sea ice revealing that the cloud coverage over the melted area remains a constant of about 81%. The polar area has components of a melted portion, frozen portion and a portion in melting or freezing. Melting or freezing areas with SIC values in the range from 1 to 0.85 may have different cloud coverage. The average cloud fractions over the sensitive zone are about 82% to 85%, which are slightly higher than the cloud fraction over the melted area. The open surface area is expected to have more clouds than the freezing up area. Most probably, melting or freezing areas have the highest cloud coverage. For the melted area in the sensitive zone, it is evident that the melted area has a cloud fraction of 81%. This huge cloud coverage over the melted or melting dark water surface can also moderate the effects of albedo feedback. Both the large cloud fraction and the response of clouds to the melting of sea ice can contribute to the smaller TOA albedo change relative to the clear sky.

The new findings give the areal distribution of the cloud response to variation in SIC. The covariance between cloud cover and sea ice is evident and statistically significant in portions of the Arctic. Based on the change of sea ice and clouds, we provided an observational radiative kernel of sea ice albedo feedback for the first time. These new findings may also improve our ability to correctly capture feedbacks in climate models and lead to more accurate simulations of climate in the Arctic, and its impact on the global climate affecting agriculture productivity^32^, air quality^33^, and the occurrence of extreme weather events^34–36^. Potentially, this cloud damping effect mechanism can enlighten the study of “white Arctic versus blue Arctic” concerning diverging stakeholder responses to environmental change^37^.

## Method

The synthesized information on radiation, cloud and surface conditions is investigated over the summer and early fall to examine the response of clouds to the sea ice retreat and to quantify the response. The joint description of the radiation field and cloud information is based on an integrated CERES, CALIPSO, CloudSat, and MODIS merged (CCCM) dataset. These data were obtained from the NASA Langley Research Center Atmospheric Science Data Center (ASDC). The advantages and the accuracy of the cloud and radiation products in the CCCM dataset were discussed by Kato and co-workers^29,38,39^. The dataset covers the period from July 1, 2006 through April 30, 2011. The data from different platforms are collocated at the CERES 20-km near-nadir footprint. Radiation information used in this paper is provided by the CERES Single Scanner Footprint TOA/Surface Fluxes (SSF) dataset, including the shortwave irradiance (0.3–5 *μ*m), Earth emitted longwave irradiance (4–40 *μ*m).

Cloud information is obtained from joint CALIPSO and CloudSat observations. The CALIOP laser deployed on CALIPSO is sensitive to ice particles^40,41^, while the Cloud Profiling Radar (CPR) deployed on CloudSat, is sensitive to (liquid and ice) water particles. The sky is regarded to be overcast when a cloud is detected by CALIPSO or CloudSat.

Based on observations of cloud fraction and irradiances, we can calculate the TOA albedo and the cloud radiative forcing. The net irradiance at frequency *ν* is defined in terms of energy per unit area per unit time as^42^:

2 $${F}_{\nu }={F}_{\nu }^{+}-{F}_{\nu }^{-}={\int }_{4\pi }\,d\omega \,\cos \,\theta {I}_{\nu }\,[{\rm{W}}\cdot {{\rm{m}}}^{-2}\cdot {{\rm{Hz}}}^{-1}]$$

where *I*_*ν*_ [${\rm{W}}\cdot {{\rm{m}}}^{-2}\cdot {{\rm{Hz}}}^{-1}\cdot {\rm{sr}}$] is the radiance. Integrating over the shortwave or longwave range we obtain the net irradiance ${F}_{sw,lw}\equiv {\int }_{sw,lw}\,d\nu {F}_{\nu }$, where *sw* and *lw* stand for the shortwave and longwave spectral ranges, respectively. In terms of the cloud fraction $\eta$, the net irradiance can be expressed as: ${F}_{sw,lw}=(1-\eta ){F}_{sw,lw}^{cl}+\eta {F}_{sw,lw}^{ov}$, where *F*^*cl*^ is the clear sky irradiance, and *F*^*ov*^ is the cloudy (overcast) sky irradiance. The cloud radiative forcing is expressed as: $C=F-{F}^{cl}=\eta ({F}^{ov}-{F}^{cl})$.

The albedo is calculated by:

3 $$\rho =\frac{{F}^{+}}{{F}^{s}\,\cos ({\theta }_{0})}$$

where *F*^+^ is the spectrally integrated upward shortwave irradiance at the TOA, *F*^*s*^ is the incoming solar irradiance (normal to the beam)) at TOA, and *θ*_0_ is the solar zenith angle.

The status of sea surface is represented by the SIC and sea ice extent. SIC is defined by the percentage of the area that is covered by the sea ice at a given point in the ocean. 0 means totally open water and 1 means totally covered by the sea ice. Sea ice extent means the total area that has SIC greater than 85% in the unit of Mkm^2^. The SIC data used in this paper are obtained from Nimbus-7 SMMR and DMSP SSM/I-SSMIS Passive Microwave observations available at the National Snow and Ice Date Center (NSIDC)^43^. The SIC data have a resolution of 25 × 25 km with the EASE-Grid 2.0 projection. The grid data are available at NSDIC. When the surface has a SIC value greater than 85%, the surface grid box is regarded to be sea ice covered. The sea ice extent is obtained from the Multisensor Analyzed Sea Ice Extent - North Hemisphere (MASIE-NH) daily value dataset, which is also available at the NSIDC.

All irradiances, albedo values, and cloud fractions are gridded to the average on the EASE-Grid 2.0 25 km grids^44^. For each grid point, we average the data over a 3° (latitude) × 8° longitude box and over a period of nine days to reduce the sampling uncertainty and smooth out the character of weather since most of the weather processes are short than nine days. Then an average over the investigated period is used to represent the value of the year. By subtracting the 4-year (2007–2010) areal average from the value of the year, we get the anomaly distribution.

The CALIPSO level 2 cloud layer product with 5 km resolution (version 3) is used to verify the response of clouds to the retreat of sea ice on a decadal time scale (from 2006 to 2015). The data are available at ASDC. The average cloud fraction is calculated over the sensitive zone which is defined in Fig. 5. The mean sea ice extent over the August 15 to October 15 period is the average of the daily sea ice extent. Because of the lag related to the formation of evaporation, we use the average cloud fraction over August to October period. The cloud-covered area is defined as the average cloud fraction on the basis of 25 km × 25 km pixels. A pixel is labeled as cloudy if the average cloud fraction is greater than 80%. The melted area is defined through the average of sea ice concentration of the basis of pixels in the sensitive zone. A pixel is labeled as melted if it has sea ice concentration smaller than 15%. Thees averaging periods and definitions of cloud-covered area and melted area are applied in the calculations in Fig. 6.

The data are being detrended by subtracting a linear fitting to remove the longtern trend in the process of time series and regression relation in Fig. 6. The CALIPSO level 2 cloud product is used in the subsection “the anomaly of cloud and sea ice concentration” and the subsection “the cloud fraction over the melted portion of Arctic Ocean”. The CCCM dataset which contains the radiation observations is used in subsections “The effects on the TOA albedo” and “Radiative effects at TOA”.

In Figs 1, 2 and 5, SIC values from July 1 to September 15 is used to do the area distribution analysis. The SIC average period mostly stands for the sea ice coverage over the melting season. After September 15 the Arctic may freeze up again. In Fig. 6, the mean sea ice extent between August 15 and October 15 are used to define the minimum sea ice extent of the year. This period covers the minimum of sea ice extent but also contains sea ice information for a period.

## References

[1] Winton Michael (2013). Sea Ice-Albedo Feedback and Nonlinear Arctic Climate Change. Arctic Sea Ice Decline: Observations, Projections, Mechanisms, and Implications.

[2] Hall Alex (2004). The Role of Surface Albedo Feedback in Climate. Journal of Climate.

[3] Pithan Felix, Mauritsen Thorsten (2014). Arctic amplification dominated by temperature feedbacks in contemporary climate models. Nature Geoscience.

[4] Serreze MC, Francis JA (2006). The arctic amplification debate. Climatic Change.

[5] Taylor PC (2013). A decomposition of feedback contributions to polar warming amplification. Journal of Climate.

[6] Pistone, K., Eisenman, I. & Ramanathan, V. Observational determination of albedo decrease caused by vanishing arctic sea ice. *Proceedings of the National Academy of Sciences* **111**, 3322–3326, http://www.pnas.org/content/111/9/3322 (2014). 10.1073/pnas.1318201111 PMC3948279 24550469

[7] Yang Xiao-Yi, Fyfe John C., Flato Gregory M. (2010). The role of poleward energy transport in Arctic temperature evolution. Geophysical Research Letters.

[8] Screen James A., Simmonds Ian (2010). The central role of diminishing sea ice in recent Arctic temperature amplification. Nature.

[9] Hansen, J. & Nazarenko, L. Soot climate forcing via snow and ice albedos. *Proceedings of the National Academy of Sciences* **101**, 423–428, http://www.pnas.org/content/101/2/423 (2004). 10.1073/pnas.2237157100 PMC327163 14699053

[10] Serreze, M. C., Barrett, A. P., Stroeve, J. C., Kindig, D. N. & Holland, M. M. The emergence of surface-based arctic amplification. *The Cryosphere* **3**, 11–19, https://www.the-cryosphere.net/3/11/2009/ (2009).

[11] Francis JA, Hunter E (2006). New insight into the disappearing arctic sea ice. Eos, Transactions American Geophysical Union.

[12] Comiso, J. C., Parkinson, C. L., Gersten, R. & Stock, L. Accelerated decline in the arctic sea ice cover. *Geophysical Research Letters* **35**, 10.1029/2007GL031972 (2008).

[13] Comiso JC, Meier WN, Gersten R (2017). Variability and trends in the arctic sea ice cover: Results from different techniques. Journal of Geophysical Research: Oceans.

[14] Soden BJ (2008). Quantifying climate feedbacks using radiative kernels. Journal of Climate.

[15] Higgins, M. E. & Cassano, J. J. Impacts of reduced sea ice on winter arctic atmospheric circulation, precipitation, and temperature. *Journal of Geophysical Research: Atmospheres* **114**, n/a–n/a, 10.1029/2009JD011884, D16107 (2009).

[16] Kopec, B. G., Feng, X., Michel, F. A. & Posmentier, E. S. Influence of sea ice on arctic precipitation. *Proceedings of the National Academy of Sciences* **113**, 46–51, http://www.pnas.org/content/113/1/46 (2016). 10.1073/pnas.1504633113 PMC4711856 26699509

[17] Taylor PC, Kato S, Xu K-M, Cai M (2015). Covariance between arctic sea ice and clouds within atmospheric state regimes at the satellite footprint level. Journal of Geophysical Research: Atmospheres.

[18] Kay JE, L’Ecuyer T (2013). Observational constraints on arctic ocean clouds and radiative fluxes during the early 21st century. Journal of Geophysical Research: Atmospheres.

[19] Perovich, D. K., Grenfell, T. C., Light, B. & Hobbs, P. V. Seasonal evolution of the albedo of multiyear arctic sea ice. *Journal of Geophysical Research: Oceans* **107**, SHE 20–1–SHE 20–13, 10.1029/2000JC000438, 8044 (2002).

[20] Donohoe A, Battisti DS (2011). Atmospheric and surface contributions to planetary albedo. Journal of Climate.

[21] Pachauri, R. K. *et al*. *Climate change 2014: synthesis report*. *Contribution of Working Groups I*, *II and III to the fifth assessment report of the Intergovernmental Panel on Climate Change* (IPCC, 2014).

[22] Kay, J. E. & Gettelman, A. Cloud influence on and response to seasonal arctic sea ice loss. *Journal of Geophysical Research: Atmospheres* **114**, 10.1029/2009JD011773, D18204 (2009).

[23] Mülmenstädt J, Lubin D, Russell LM, Vogelmann AM (2012). Cloud properties over the north slope of alaska: Identifying the prevailing meteorological regimes. Journal of Climate.

[24] Liu Z, Schweiger A (2017). Synoptic conditions, clouds, and sea ice melt onset in the beaufort and chukchi seasonal ice zone. Journal of Climate.

[25] Morrison AL, Kay JE, H. Chepfer RG, Yettella V (2018). Isolating the liquid cloud response to recent arctic sea ice variability using spaceborne lidar observations. Journal of Geophysical Research: Atmospheres.

[26] Abe, M., Nozawa, T., Ogura, T. & Takata, K. Effect of retreating sea ice on arctic cloud cover in simulated recent global warming. *Atmospheric Chemistry and Physics Discussions* **15**, 17527–17552, https://www.atmos-chem-phys-discuss.net/15/17527/2015/ (2015).

[27] Liu, Y. & Key, J. R. Less winter cloud aids summer 2013 arctic sea ice return from 2012 minimum. *Environmental Research Letters* **9**, 044002, http://stacks.iop.org/1748-9326/9/i=4/a=044002 (2014).

[28] Cox CJ (2016). The role of springtime arctic clouds in determining autumn sea ice extent. Journal of Climate.

[29] Liu, Y., Key, J. R., Ackerman, S. A., Mace, G. G. & Zhang, Q. Arctic cloud macrophysical characteristics from cloudsat and calipso. *Remote Sensing of Environment* **124**, 159–173, http://www.sciencedirect.com/science/article/pii/S003442571200209X (2012).

[30] Kwok R., Rothrock D. A. (2009). Decline in Arctic sea ice thickness from submarine and ICESat records: 1958-2008. Geophysical Research Letters.

[31] Wang, X. & Key, J. R. Recent trends in arctic surface, cloud, and radiation properties from space. *Science* **299**, 1725–1728, http://science.sciencemag.org/content/299/5613/1725 (2003). 10.1126/science.1078065 12637742

[32] Kim Jin-Soo, Kug Jong-Seong, Jeong Su-Jong, Huntzinger Deborah N., Michalak Anna M., Schwalm Christopher R., Wei Yaxing, Schaefer Kevin (2017). Reduced North American terrestrial primary productivity linked to anomalous Arctic warming. Nature Geoscience.

[33] Wang, H.-J. & Chen, H.-P. Understanding the recent trend of haze pollution in eastern china: roles of climate change. *Atmospheric Chemistry and Physics* **16**, 4205–4211, https://www.atmos-chem-phys.net/16/4205/2016/ (2016).

[34] Francis Jennifer A., Vavrus Stephen J. (2012). Evidence linking Arctic amplification to extreme weather in mid-latitudes. Geophysical Research Letters.

[35] Cohen Judah, Screen James A., Furtado Jason C., Barlow Mathew, Whittleston David, Coumou Dim, Francis Jennifer, Dethloff Klaus, Entekhabi Dara, Overland James, Jones Justin (2014). Recent Arctic amplification and extreme mid-latitude weather. Nature Geoscience.

[36] Parkinson CL, Comiso JC (2013). On the 2012 record low arctic sea ice cover: Combined impact of preconditioning and an august storm. Geophysical Research Letters.

[37] Newton R (2016). White arctic vs. blue arctic: A case study of diverging stakeholder responses to environmental change. Earth’s Future.

[38] Kato, S. *et al*. Improvements of top-of-atmosphere and surface irradiance computations with calipso-, cloudsat-, and modis-derived cloud and aerosol properties. *Journal of Geophysical Research: Atmospheres* **116**, 10.1029/2011JD016050, D19209 (2011).

[39] Kato, S. *et al*. Relationships among cloud occurrence frequency, overlap, and effective thickness derived from calipso and cloudsat merged cloud vertical profiles. *Journal of Geophysical Research: Atmospheres* **115**, 10.1029/2009JD012277 (2010).

[40] Hu Yongxiang, Vaughan Mark, Liu Zhaoyan, Lin Bing, Yang Ping, Flittner David, Hunt Bill, Kuehn Ralph, Huang Jiangping, Wu Dong, Rodier Sharon, Powell Kathy, Trepte Charles, Winker David (2007). The depolarization - attenuated backscatter relation: CALIPSO lidar measurements vs. theory. Optics Express.

[41] Hu Y (2009). Calipso/caliop cloud phase discrimination algorithm. Journal of Atmospheric and Oceanic Technology.

[42] Stamnes, K. & Stamnes, J. *Radiative Transfer in Coupled environmental system* (John Wiley & Sons, 2017).

[43] Cavalieri, D. J., Parkinson, C. L., Gloersen, P. & Zwally, H. J. 1996, updated yearly. sea ice concentrations from nimbus-7 smmr and dmsp ssm/i-ssmis passive microwave data, version 1.[north hemisphere, daily]. *Boulder*, *Colorado USA*. *NASA National Snow and Ice Data Center Distributed Active Archive Center* (updated yearly, 1996).

[44] Brodzik, M. J., Billingsley, B., Haran, T., Raup, B. & Savoie, M. H. Ease-grid 2.0: Incremental but significant improvements for earth-gridded data sets. *ISPRS International Journal of Geo-Information* **1**, 32–45, http://www.mdpi.com/2220-9964/1/1/32 (2012).

